# Clinicopathologic Significance of HIF-1*α*, CXCR4, and VEGF Expression in Colon Cancer

**DOI:** 10.1155/2010/537531

**Published:** 2010-10-07

**Authors:** Yugang Wu, Min Jin, Huanbai Xu, Zhang Shimin, Songbing He, Liang Wang, Yanyun Zhang

**Affiliations:** ^1^Department of Surgery, The First People Hospital of Changzhou and The Third Affiliated Hospital of Soochow University, Changzhou, Jiangsu 213000, China; ^2^Key Laboratory of Stem Cell Biology, Institute of Health Sciences, Shanghai Institutes for Biological Sciences and Shanghai Jiao Tong University School of Medicine, Chinese Academy of Sciences, Shanghai 200025, China; ^3^Department of Surgery, The First Affiliated Hospital of Soochow University, Suzhou, Jiangsu 215006, China

## Abstract

We investigated the clinicopathologic significance of HIF-1, CXCR4, and VEGF expression using immumohistochemistry in human colon cancer. HIF-1, CXCR4, and VEGF high expression levels were correlated positively with TNM stage, lymph node involvement, and distant metastasis Furthermore, we found that combined high expression of any two of the three molecules (*P* = .028 for HIF-1/CXCR4, *P* = .007 for HIF-1/VEGF, and *P* = .004 for CXCR4/VEGF) had stronger correlation with lymph node metastasis than did each alone. However, a relationship with distant metastasis is seen only with the combinations CXCR4/VEGF (*P* = .069 for HIF-1/CXCR4, *P* = .062 for HIF-1/VEGF, and *P* = .035 for CXCR4/VEGF) as compared with those of single molecule high expression alone. Combined expression of all three molecules strongly correlates with lymph node metastasis and distant metastasis. The mRNA expression of HIF-1, CXCR4, and VEGF were quantified by real-time PCR in different colon cancer tissue samples, the experiment results shown that fresh colon tissue samples significantly overexpressed CXCR4 and VEGF mRNA compared with negative control. Therefore, the disease-free survival of all patients after curative resection can be considered in association with all three markers expression.

## 1. Introduction

Colon cancer is one of the most common cancers frequently metastasizing to the liver, lymph nodes, and peritoneum [1]. Currently, radical surgery represents the standard method of therapy. Adjuvant therapy such as chemotherapy and radiation therapy have been widely applied, but colon cancer control at the advanced stage remains difficult [2]. The 5-year survival rate for patients with the spread of disease to distant sites is approximately 19% [3]. Therefore, it is necessary to evaluate whether some metastasis-related molecules can be used as prognostic markers for metastasis of colon cancer.

Hypoxia-inducible factor-1 (HIF-1) is a heterodimeric basic helix-loop-helix transcription factor composed of two subunits, HIF-1*α* and HIF-1*β* [4]. HIF-1*α* is the key regulatory component because it is degraded rapidly in normoxic conditions but is stabilized and activated during hypoxia and also is one of the key factors promoting carcinogenesis independent of histogenetic origin [5]. In clinical samples, HIF-1*α* is found elevated and correlates with tumor progression, aggressive behavior, and patient prognosis in several types of carcinoma including those of the ovary, breast, prostate, lung, renal, glial, and melanoma [6]. 

HIF-1 has emerged as a critical regulator of the cellular response to hypoxia since it is ubiquitously expressed and induces the expression of many hypoxia-inducible genes (HRE) [7]. A gene reported to be positively regulated by HIF-1*α* is CXC chemokine receptor 4 (CXCR4) [8]. Chemokines comprise a superfamily of small cytokines with the ability to chemoattract cells to target tissues. Interactions between CXCR4 and its ligand CXCL12 (stromal cell-derived factor 1, SDF-1) play an important role in the directional regulation of hematopoiesis, migration of hematopoietic cells, angiogenesis, and migration of metastatic tumor cells [9]. CXCR4 is the most common chemokine expressed in human tumors such as breast cancer, colorectal cancer, and ovarian cancer, and SDF-1 is highly expressed at sites of metastasis including the lung, bone marrow, lymph nodes, and liver [10]. Studies have shown that HIF-1*α* is a potent inducer of both CXCR4 and SDF-1 expression in a variety of cell types [11, 12].

Angiogenesis is known to play an important role in the development of tumor growth and metastasis. Vascular endothelial growth factor (VEGF) is the most important and best characterized angiogenic factor [13]. VEGF potently increases vascular permeability and promotes the formation of new blood vessels by stimulating endothelial cells to migrate and divide [14]. HIF can directly activate the expression of a number of proangiogenic factors. Of all those induced by HIF, VEGF is particularly noteworthy since it has potent angiogenic properties and is expressed in a large number of human tumors [15, 16]. The prognostic value of overexpression of VEGF has been demonstrated in many types of solid human cancers. Based on these studies, we suggest that combinations of HIF-1*α*, CXCR4, and VEGF expression in tumor tissue will be useful for predicting clinicopathologic significance and tumor metastasis.

In this study, we demonstrate high expression of HIF-1*α*, CXCR4, and VEGF in human colon cancer specimens using immunohistochemistry. Furthermore, we investigate whether the expression of HIF-1*α*, CXCR4, and VEGF have a significant correlation with clinicopathologic factors of colon cancer. The mRNA expression of HIF-1*α*, CXCR4, and VEGF in colon cancer were quantified by real-time PCR.

## 2. Materials and Methods

### 2.1. Patients and Specimens

Colon cancer samples were collected from 68 patients undergoing curative-intent surgery at the Department of General Surgery of the First People's Hospital of Changzhou (Jiangsu, China) from 2006 to 2008. Colon hyperplastic polyp tissue (HPP) samples were obtained from 8 patients undergoing colonoscopy with biopsy extraction at the Digestion Internal Medicine Department during the years 2006–2008. There were also 10 normal colonic tissue samples adjacent to colon tumor (used as controls). The histologic sections were reviewed by two expert pathologists to verify the histologic diagnosis. None of the patients had received any preoperative treatment. The clinicopathologic characteristics of these patients are shown in Table 1. Tumors were staged according to the American Joint Committee on Cancer (AJCC) pathologic tumor-node-metastasis (TNM) classification. Informed consent was obtained from all study subjects before sample collection and these samples were used according to ethical standards.

### 2.2. Immunohistochemistry

Immunohistochemistry was performed on the primary tumors as previously described [17]. Sections were subjected to routine deparaffinization and rehydration. Antigen retrieval was achieved by microwaving in 0.01 mol/L citrate buffer for 10 mins and then cooling for 30 min. Endogenous peroxidase activity was inhibited by incubation with 3% hydrogen peroxide in methanol for 20 min and nonspecific binding was blocked by incubation with 5% bovine serum albumin in phosphate-buffered saline (PBS) at room temperature (RT). After three PBS washes, the specimens were reacted overnight at 4°C with murine antihuman monoclonal antibodies: anti-HIF-1*α* (diluted 1 : 200, Clone H1apha67, ABCam), anti-CXCR4 (Clone 44716, R&D Systems, Inc., Minneapolis, MN, USA), or anti-VEGF (Clone JH121, NeoMarkers, Lab Vision Corporation, Fremont, CA, USA). After incubation with rat anti-mouse-IgG2b-horseradish peroxidase, signal was developed with 3,3 –diaminobenzidine tetrahydrochloride in Tris–HCl buffer (pH 7.6) containing 0.02% hydrogen peroxide. The sections were then counterstained with hematoxylin and mounted. Negative controls were performed by replacing the specific primary antibody with PBS.

### 2.3. Histological Assessment

Two clinical pathologists independently evaluated the immunostaining results. When an evaluation differed, the final decision was made by consensus. Specific immunoreactivity was observed in the cytoplasm and in nuclei of tumor cells. Cytoplasmatic staining was observed homogeneously in the tumor cells; nuclear immunoreactivity was heterogeneous in the tumor, so the score was ascertained by consideration of both staining density and intensity. The extent of the staining was categorized into five semiquantitative classes based on the percentages of positive tumor cells: 0 (<5% positive cells), 1 (6–25% positive cells), 2 (26–50% positive cells), 3 (51–75% positive cells), and 4 (>75% positive cells). The intensity of staining was also determined semiquantitatively on a scale of 0–3 as follows: 0 (negative), 1 (weakly positive), 2 (moderately positive), and 3 (strongly positive). Multiplication of the intensity and the percentage scores gave rise to the final staining score: 0 (negative), + (1–4), ++ (5–8), and +++ (9–12). For statistical analysis, tumors having a final staining score of negative or +, which showed a weak or moderate/strong immunoreactivity were combined into a low expression group and were compared to tumors with scores of ++ or +++ as the high expression group.

### 2.4.. Real-Time PCR

The mRNA expression of HIF-1*α*, CXCR4, and VEGF were quantified by real-time PCR in different fresh samples, including normal colonic tissue samples (*n* = 6, negative control group), colon cancer with lymph node metastasis samples (*n* = 6, lymph node metastasis group), and colon cancer with distant metastasis samples (*n* = 6, distant metastasis group). Total RNA was isolated using TRIzol (Invitrogen, Carlsbad, CA) according to the manufacturer's instructions, and reverse transcribed. mRNA expression of HIF-1*α*, CXCR4, VEGF, and *β* actin was determined by real-time PCR using SYBR Green master mix (ABI, Foster City, CA). The primers for HIF-1*α* were 5′-GCTTGCTCATCAGTTGCCAC-3′and 5′-CATAACAAAACCATCCAAGGC-3′. The primers for CXCR4 were 5′-GCATGACGGACAAGTACAGGCT-3′ and 5′-AAAGTACCAGTTTGCCACGGC-3′. The primers for VEGF were 5′-CAACATCACCATGCAGATTATGC-3′ and 5′-TCGGCTTGTCACATTTTTCTTGT-3′. The primers for *β* actin were 5′-ATGGAGGGGAATACAGCCC-3′ and 5′-TTCTTTGCAGCTCCTTCGTT-3′. Data were collected and quantitatively analyzed on an ABI Prism 7900 sequence detection system (ABI). *β*-actin gene was used as an endogenous control for sample normalization. Differences in the relative level of three markers normalized to *β* actin can be estimated by differences in the ratio.

### 2.5. Statistical Analysis

Differences were evaluated using Statistical Package for Social Science software (version 11.0, SPSS Inc., Chicago, IL). The association of staining intensity with clinicopathologic patterns was assessed with Chi-squared test and two-sided Fisher's exact test to determine the significance of the difference between the the covariates. Differences in mRNA expression levels were evaluated with Student's *t*-test. Statistical tests were two sided. *P*  values <.05 were considered to be statistically significant. 

## 3. Results

### 3.1. Patient Characteristics

A total of 68 patients with colon cancer were included in the current analyses (Table 1). The group was comprised of 41 males and 27 females, with a median age of 63 years (range, 39–81 years). Forty one patients (60%) evidenced tumor sizes of ≥5 cm. The postoperative stages of patients were I, II, III, and IV in 6, 30, 25, and 7 patients, respectively. Thirty two patients (47%) had lymph node metastasis. Seven patients (10%) had distant metastasis. 

### 3.2. HIF-1*α*, CXCR4, and VEGF Immunostaining in Different Colon Lesions

**Figure 1:**
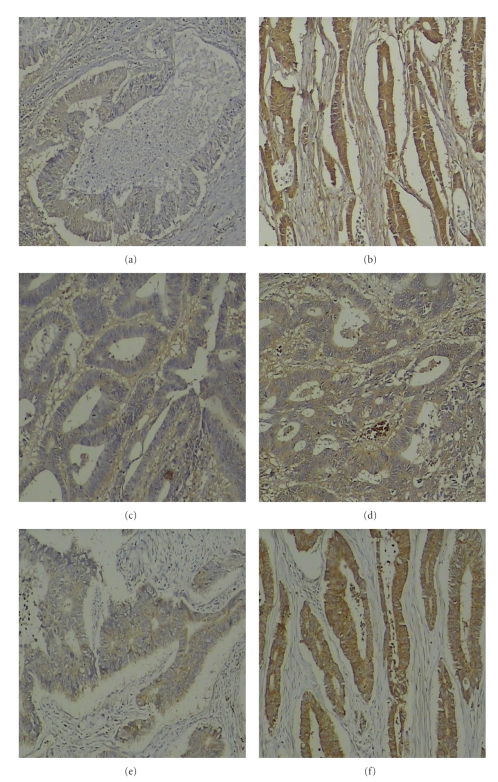
Expression of HIF-1*α*, CXCR4, and VEGF in colon primary tumor samples. Sections were subjected to routine deparaffinization and rehydration. Antigen retrieval was achieved by microwaving in citrate buffer for 10 min. The endogenous peroxidase activity was inhibited by incubation with 3% hydrogen peroxide. The specimens were reacted overnight with anti-HIF-1*α* antibodies, anti-CXCR4 antibodies, and anti-VEGF antibodies, then were incubated with rat anti-mouse-IgG2b-horseradish peroxidase. The sections were then counterstained with hematoxylin and mounted (see Section 2). HIF-1*α* staining was observed in the cytoplasm and in the nucleus of the tumor cells, CXCR4, and VEGF staining was observed in the cytoplasm of tumor cells. Positive staining was observed as a brown color. Weakly positive immunohistochemical staining of three molecules in (a) (HIF-1*α*), (c) (CXCR4), (e) (VEGF), respectively (×100). Strongly positive immunohistochemical staining of three molecules in (b) (HIF-1*α*), (d) (CXCR4), (f) (VEGF), respectively, (×100).

**Figure 2:**
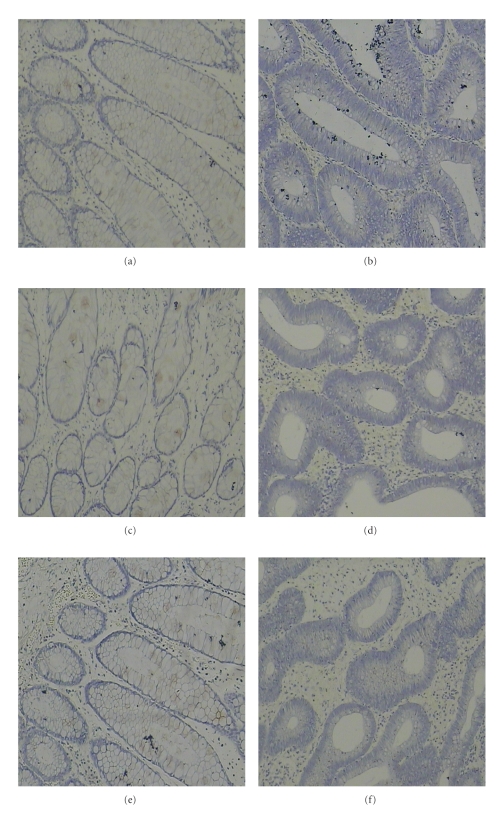
Expression of HIF-1*α*, CXCR4, and VEGF in normal colonic tissue and hyperplastic polyps (HPP). HIF-1*α*, CXCR4, and VEGF staining was negative in normal colonic tissue and HPP. Negative immunohistochemical staining of normal colonic tissue in (a) (HIF-1*α*), (c) (CXCR4), (e) (VEGF), respectively (×100). Negative immunohistochemical staining of HPP in (b) (HIF-1*α*), (d) (CXCR4), (f) (VEGF), respectively, (×100).

HIF-1*α*, CXCR4, and VEGF immunostaining was quantitatively assessed and grouped into high- or low-grade categories. Immunohistochemical expression of HIF-1*α* was observed in the cytoplasm and in the nucleus of the tumor cells in colon cancer cases but with different staining intensities (Figures 1(a) and 1(b)). Normal colonic mucosa and HPP showed neither specific cytoplasmic nor nuclear HIF-1*α* expression (Figures 2(a) and 2(b)). However, 30 colon cancer cases (44%) showed high HIF-1*α* expression (Figure 1(b)). There was no significant correlation in the cytoplasmic or nuclear expression between nonmetastatic tumors and carcinomas with lymph node and distant metastases. Immunohistochemical expression of CXCR4 was observed predominantly in the cytoplasm of tumor cells (Figures 1(c) and 1(d)). All ten normal colonic mucosa cases and 8 HPP cases showed very weak CXCR4 staining or no staining (Figures 2(c) and 2(d)). 41 colon cancer cases (60%) showed high CXCR4 expression (Figure 1(d)). VEGF immunoreactivity was observed in the cytoplasm of neoplastic cells in all investigated colon cancer cases (Figures 1(e) and 1(f)). In colon cancer cases, high VEGF expression was observed in 44 (65%) cases by immunohistochemistry (Figure 1(f)). In comparison, VEGF staining of normal colonic mucosa cases and HPP cases was negative (Figures 2(e) and 2(f)). The differences in expression of the three molecules (HIF-1*α*, CXCR4, and VEGF) between benign hyperplastic polyps and colon cancer tissues were all found to be statistically significant (*P* < .05; Table 1).

### 3.3. Clinicopathological Significance of HIF-1*α*, CXCR4, and VEGF Expression

The relationships between HIF-1*α*, CXCR4, and VEGF protein expression levels and clinicopathologic variables are provided in Table 1. The high expression rates of HIF-1*α*, CXCR4, and VEGF were 44%, 60%, and 65%, respectively. HIF-1*α*, CXCR4, and VEGF expression levels were correlated positively with TNM stage (*P* = .001, *P* = .003, *P* = .008, resp.). There were no statistically significant differences in these molecules (HIF-1*α*, CXCR4, and VEGF) with regard to patient age (*P* = .878, *P* = .801, *P* = .195, resp.), sex (*P* = .587, *P* = .639, *P* = .807, resp.), or tumor size (*P* = .649, *P* = .297, *P* = .  0340, resp.). 

### 3.4. Association of Single or Combined HIF-1*α*, CXCR4, and VEGF High Expression with Lymph Node Metastasis

Statistical analysis shows that the incidence of lymph node metastasis tends to be higher in patients with colon cancer with high rather than low expression of HIF-1*α*, CXCR4, or VEGF (*P* < .001, *P* = .001, *P* = .001, resp.; Table 1). We further compared correlation of lymph node metastasis of colon cancer with combined high expression of both HIF-1*α* and CXCR4, of both HIF-1*α* and VEGF, or of both CXCR4 and VEGF. Our data show that the incidence of lymph node metastasis is significantly higher (74%) in patients with tumors highly expressing both HIF-1*α* and CXCR4 than in those patients (43%) with tumors highly expressing only one of the molecules, or in those patients (11%) with tumors not expressing either of these two molecules. Similar results were observed in patients with tumors highly expressing both HIF-1*α* and VEGF, or both CXCR4 and VEGF. At the same time, there were statistically significant differences in high expression of these single molecules, and high expression of any two of the three molecules with regard to lymph node metastasis in patients with colon cancer (*P* = .028 for HIF-1*α*/CXCR4, *P* = .007 for HIF-1*α*/VEGF, and *P* = .004 for CXCR4/VEGF; Table 2). In addition, as shown in Table 2 out of the 32 cases with lymph node metastasis, 18 cases showed high expression of all HIF-1*α*, CXCR4, and VEGF molecules. 14 cases did not show high expression in all three markers. Statistical analysis showed that combined high expression of all three molecules (HIF-1*α*, CXCR4, and VEGF) is significantly associated with lymph node metastasis in patients with colon cancer as compared with cases not showing such expression (*P* < .001; Table 2).

### 3.5. Association of Single or Combined HIF-1*α*, CXCR4, and VEGF High Expression with Distant Metastasis

As shown in Table 1 the incidence of distant metastasis tends to be higher in patients with colon cancer with high rather than low expression of HIF-1*α*, CXCR4, or VEGF (*P* = .019, *P* = .037, *P* = .046, resp.). Moreover, experimental data show that there are statistically significant differences in high expression of single CXCR4 or VEGF molecules, and high expression of both CXCR4 and VEGF molecules with regard to distant metastasis in patients with colon cancer (*P* = .035 for CXCR4/VEGF; Table 2). However, this result does not apply in the case of any other combination of two of the three molecules (*P* = .069 for HIF-1*α*/CXCR4, and *P* = .062 for HIF-1*α*/VEGF; Table 2). We found a correlation of distant metastasis of colon cancer with combined high expression of all three HIF-1*α*, CXCR4, and VEGF markers. Out of the 7 cases with distant metastasis, 6 cases showed high expression of all HIF-1*α*, CXCR4, and VEGF molecules; 1 case showed high expression in fewer than three markers. Statistical analysis indicates that combined high expression of all three molecules is significantly associated with distant metastasis in patients with colon cancer as compared with cases of high expression in fewer than all three (*P* < .001; Table 2).

### 3.6. The mRNA Expression of HIF-1*α*, CXCR4, and VEGF in Normal Colonic Tissues and Colon Cancer Tissues

**Figure 3:**
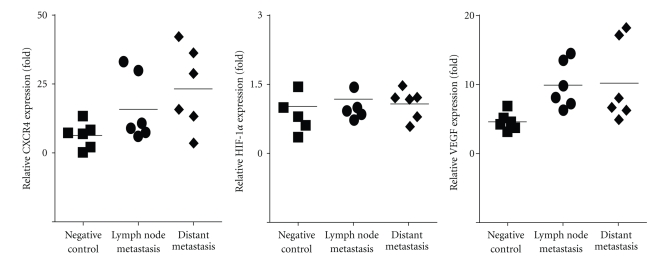
Expression of HIF-1*α*, CXCR4, and VEGF mRNA in normal colon tissues and colon cancer. The mRNA expression of HIF-1*α*, CXCR4, and VEGF were quantified by real-time PCR in different samples, including normal colonic tissue samples (*n* = 6, negative control group), colon cancer with lymph node metastasis samples (*n* = 6, lymph node metastasis group), and colon cancer with distant metastasis samples (*n* = 6, distant metastasis group). Total RNA was isolated using TRIzol according to the manufacturer's instructions and reverse transcribed. mRNA expression of HIF-1*α*, CXCR4, VEGF and *β* actin was determined by real-time PCR using SYBR Green master mix (see Section 2). Differences in the relative level of three markers normalized to *β* actin can be estimated by differences in the ratio. CXCR4, 15.91 ± 12.14-fold in lymph node metastasis group or 23.20 ± 14.84-fold in distant metastasis group versus 6.23 ± 4.66-fold in negative control group, respectively, *P* < .05, VEGF, 9.99 ± 3.38-fold in lymph node metastasis group or 10.27 ± 5.90-fold in distant metastasis group versus 4.68 ± 1.28-fold in negative control, respectively, *P* < .05.

In order to quantify the message RNA of these marks in colon cancer, expression of HIF-1*α*, CXCR4, and VEGF at the mRNA levels was analyzed by real-time PCR in different samples. Results demonstrated that mean HIF-1*α* mRNA expression levels were 1.05 ± 0.58-fold in negative control group, 1.19 ± 0.52-fold in lymph node metastasis group, and 1.09 ± 0.32-fold in distant metastasis group. There was no statistically significant difference in HIF-1*α* mRNA expression between tumor groups and control group. In contrast, CXCR4 mRNA expression levels were significantly higher in lymph node metastasis group (15.91 ± 12.14 fold) and distant metastasis group (23.20 ± 14.84 fold) than in normal colonic tissue (6.23 ± 4.66 fold). Statistical analysis has shown that there was a significant difference between the mean CXCR4 mRNA levels for all groups (lymph node metastasis group or distant metastasis group *vs* negative control group, resp., *P* < .05, Figure 3). In addition, lymph node metastasis group and distant metastasis group significantly overexpressed VEGF mRNA compared with negative control group, respectively, (9.99 ± 3.38-fold in lymph node metastasis group or 10.27 ± 5.90-fold in distant metastasis group versus 4.68 ± 1.28-fold in negative control group, resp., *P* < .05, Figure 3).

## 4. Discussion

In this study, we investigated HIF-1*α*, CXCR4, and VEGF expression in human colon primary tumor samples using immunohistochemistry. Staining was observed in the cytoplasm or in the nucleus of tumor cells. The results demonstrate that there is a statistically significant correlation between human colon cancer TNM stage and single HIF-1*α*, CXCR4, and VEGF expression levels. We also found that high expression of HIF-1*α*, CXCR4, and VEGF is associated with increased metastatic potential in human colon cancer. Furthermore, concomitant expression of the three molecules may be considered in association with the disease-free survival of all patients after curative resection. We also investigated that expression of HIF-1*α*, CXCR4, and VEGF at the mRNA levels was analyzed by real-time PCR. The experiment results demonstrated that CXCR4 mRNA and VEGF mRNA expression levels were significantly higher in fresh colon cancer samples than in normal colonic tissue, whereas tumoral HIF-1*α* expression at the mRNA levels was not higher.

Tumor hypoxia is a microenvironmental factor that has been shown to affect the malignant progression of transformed cells [18]. In the presence of oxygen, HIF-1*α* is hydroxylated by a family of prolyl hydroxylases and subjected to rapid ubiquitination and proteasomal degradation mediated by the Von-Hippel Lindau-dependent pathway (VHL) [19, 20]. Under hypoxic conditions, hydroxylation is inhibited, resulting in the stabilization of HIF-1*α* protein, which translocates into the nucleus, dimerizes with HIF-1*α*, and activates HRE, many of which are important for proliferation, apoptosis, angiogenesis, and migration [21]. Previous studies have indicated that activation of HIF-1*α* has been identified in many solid tumors including carcinomas of the gastrointestinal tract. Our results indicated that high expression of HIF-1*α* protein is much more frequent in colon cancer tissues than in normal tissues or nontumor lesions, while the expression of HIF-1*α* significantly correlates with tumor TNM stage (*P* = .001), lymph node status (*P* < .001), and distant metastases (*P* = .019). In accordance with our results, Simiantonaki et al. [22] found that higher HIF-1*α* expression is associated with lymph node metastasis or distant metastases. 

HIF-1*α* activation correlates with metastasis and can promote metastasis through the regulation of key factors governing tumor cell metastatic potential, including E-cadherin, lysyl oxidase (LOX), connective tissue growth factor (CTGF), and plasminogen activator inhibitor-1 (PAI-1), CXCR4 [23–27]. The chemokine receptor CXCR4 selectively binds the CXC chemokine, SDF-1. Binding of SDF-1 to CXCR4 induces migration of cancer cells into normal tissue, where the cells proliferate and form metastatic tumors [28]. Schimanski et al. [29] reported in accordance with our results, that high expression of CXCR4 in colorectal cancer is significantly associated with advanced UICC tumor stages and with lymphatic or hematogenic metastasis. Recently, new evidence indicated that hypoxia may affect the tumor migration process by altering the expression of CXCR4 via activation of HIF-1*α*. Zagzag et al. [30] reported that CXCR4 expression in glioma cells can be controlled by hypoxia and the levels of HIF-1*α*, and is associated with increased glioma cell migration. Wang et al. [31] also demonstrated that exposure to hypoxia significantly enhances CXCR4 expression levels in N9 microglia cells. Blockade of HIF-1*α* activation by inhibitors of the phosphoinositide-3-kinase (PI3K)/Akt signaling pathway abrogates both hypoxia-induced CXCR4 upregulation and cell-migration acceleration. Their results point to a crucial role for the Hypoxia-HIF-1*α*-CXCR4 pathway during microglia migration. Therefore, we analyzed the correlation between the high expression of both HIF-1*α* and CXCR4 and clinicopathologic significance in human colon cancer samples. Our results show that high expression of both molecules is more predictive of lymph node involvement as compared with that of a single molecule alone. However, combined high expression of both molecules is not significantly associated with distant metastasis as compared with that of a single molecule alone (*P* = .062). 

VEGF is the most well-studied angiogenic factor. The most potent inducer of VEGF gene expression is hypoxia [32]. VEGF is transcriptionally regulated by HIF-1*α*, which translocates to the nucleus following hypoxia-induced stabilization, dimerizes with HIF-1*β* (ARNT), and activates HRE in the promoter region of the VEGF gene [33]. VEGF is also regulated by HIF-independent mechanisms including other transcription factors and coactivators [34], micro-RNAs [35], and inflammatory mediators [36]. Mizukami et al. [37] reported that VEGF may be induced by hypoxia through HIF-dependent and HIF-independent pathways, and K-ras also can induce VEGF in hypoxia independent of HIF-1 in colon cancer. These data suggest the existence of multiple mechanisms regulating the hypoxic induction of VEGF. In our study, we first demonstrated VEGF expression in human colon primary tumor tissue using immunohistochemistry. High expression of VEGF significantly correlates with tumor TNM stage (*P* = .008), lymph node status (*P* = .001), and distant metastasis (*P* = .046). Furthermore, we analyzed the concomitant expression of HIF-1*α* and VEGF in colon cancer samples, demonstrating that 63% of colon cancer with lymph node involvement showed high expression of both HIF-1*α* and VEGF, and that 6 out of 7 of the cases with both HIF-1*α* and VEGF high coexpression showed distant metastasis. Statistical analysis indicated that combined high expression of both HIF-1*α* and VEGF is significantly associated with lymph node metastasis as compared with that of a single molecule's high expression alone (*P* = .007) and cases without distant metastasis (*P* = .067). Therefore, we provide clinical evidence that coexpression of HIF-1*α* and VEGF may play an important role in colon cancer growth and lymph node metastasis.

Recently, some studies have shown that angiogenesis mediated by CXCR4 is regulated at the receptor level by VEGF. CXCL12 can induce secretion of VEGF in human arterial endothelial cells [38]. The interaction of CXCR4 and CXCL12 may promote tumor progression and metastasis via the induction of VEGF-mediated angiogenesis [39]. Therefore, we analyzed the correlation between coexpression of both molecules (CXCR4 and VEGF) and metastasis. We found a positive correlation between coexpression of both molecules and lymph node metastasis (*P* = .004) and distant metastases (*P* = .035), as compared with that of a single molecules high expression alone.

Furthermore, we analyzed the clinicopathologic significance of combined HIF-1*α*, CXCR4, and VEGF expression in colon cancer. We found that 18 out of 23 (78%) of the cases with all three molecules coexpressed showed lymph node metastasis and 6 out of 23 (26%) of the cases with all three molecules coexpressed showed distant metastasis. Statistical analysis indicates that HIF-1*α*, CXCR4, and VEGF high expression significantly correlates with lymph node status (*P* < .001) and distant metastasis (*P* < .001) as compared with cases of fewer than all three molecules showing high expression.

Lastly, in order to quantify the message RNA of HIF-1*α*, CXCR4, and VEGF in colon cancer, the three marks expression at the mRNA levels were analyzed by real-time PCR. Results demonstrated that CXCR4, and VEGF expression at the mRNA levels by real-time PCR were similar at the protein levels by immunohistochemistry in colon cancer. However, there was no statistically significant difference in HIF-1*α* mRNA expression between colon cancer group and normal colonic group. Under both normoxia and hypoxia, HIF-1*α* mRNA is expressed constitutively in many cell types, including cancer cells and endothelial cells [40]. Several putative sites for factors that activate and repress transcription were identified in the HIF1A gene promoter; some transcription factors, like Sp1, were shown to contribute to the constitutive transcription of the HIF-1*α* mRNA [41].

In summary, we have demonstrated that HIF-1*α*, CXCR4, and VEGF are highly expressed in colon cancer samples as demonstrated using immunohistochemistry. Combined high expression of any two of them is significantly associated with lymph node metastasis. However, no two of them in combination show a relationship with distant metastasis, except in the case of CXCR4/VEGF. Moreover, our data also indicate that the combined high expression of HIF-1*α*, CXCR4, and VEGF is significantly associated with lymph node metastasis and distant metastasis. These experimental results provide a possible basis for disease-free survival of all patients after curative resection to predict tumor recurrence and metastasis.

## Figures and Tables

**Table 1:** Correlation of HIF-1*α*, CXCR4, and VEGF expression with clinicopathologic features in colon cancer. Sections were subjected to routine deparaffinization and rehydration. Antigen retrieval was achieved by microwaving in citrate buffer for 10 min. The endogenous peroxidase activity was inhibited by incubation with 3% hydrogen peroxide. The specimens were reacted overnight with anti-HIF-1*α* antibodies, anti-CXCR4 antibodies, and anti-VEGF antibodies, then were incubated with rat anti-mouse-IgG2b-horseradish peroxidase. The sections were then counterstained with hematoxylin and mounted (see Section 2). Staining intensity and percentage of positive tumor cells were assessed. Multiplication of the intensity and the percentage scores gave rise to the final staining score: 0 (negative), + (1–4), ++ (5–8), and +++ (9–12). For statistical analysis, tumors having staining scores of 0 or + were designated the low expression group, and tumors with scores of ++ or +++ the high expression group. The correlation between clinicopathologic parameters and HIF-1*α*, CXCR4, and VEGF expression were analyzed by Chi-squared test or Fisher's exact test. Abbreviations: HIF-1*α*: Hypoxia-inducible factor-1; CXCR4: CXC chemokine receptor 4; VEGF: vascular endothelial growth factor; HPP: hyperplastic polyps; TNM: tumor-node-metastasis.

Clinicopathologic parameters	Case No.	HIF-1*α* expression		*P* value	CXCR4 expression		*P* value	VEGF expression		*P* value
		Low	High		Low	High		Low	High	
Total cases	68	38	30		27	41		24	44	
Age										
≤60	19	12	10	*P* = .878	8	11	*P* = .801	9	10	*P* = .195
>60	49	26	20		19	30		15	34	
Tissue type										
Normal colonic tissue	10	10	0	*P* = .002^a^	10	0	*P* < .001^a^	10	0	*P* < .001^a^
HPP	8	8	0	*P* = .006^b^	8	0	*P* < .001^b^	8	0	*P* < .001^b^
Colon cancer	68	38	30	*P* = .019^c^	27	41	*P* = .001^c^	24	44	*P* = .001^c^
Sex										
Male	41	24	17	*P* = .587	15	26	*P* = .639	14	27	*P* = .807
Female	27	14	13		11	15		10	17	
Tumor size										
≤5 cm	27	16	11	*P* = .649	13	14	*P* = .297	17	10	*P* = .340
>5 cm	41	32	19		25	16		21	20	
TNM stage										
I	6	5	1		5	1		4	2	
II	30	23	7	*P* = .001	16	14	*P* = .003	15	15	*P* = .008
III	25	9	16		6	19		5	20	
IV	7	1	6		0	7		0	7	
Lymph node metastasis										
Negative	36	28	8	*P* < .001	21	16	*P* = .001	19	17	*P* = .001
Positive	32	10	22		6	26		5	27	
Distant metastasis										
Negative	61	37	24	*P* = .019	27	34	*P* = .037	24	37	*P* = .046
Positive	7	1	6		0	7		0	7	

^a^Denotes significant difference among the three tissue types.

^b^Denotes significant difference between colon cancer and normal colonic tissue.

^c^Denotes significant difference between colon cancer and HPP.

**Table 2:** Correlation of combined high expression of HIF-1*α*, CXCR4, and VEGF with lymph node status and distant metastasis.

	Lymph node metastasis		*P* value	Distant metastasis		*P*-value
	Negative	Positive		Negative	Positive	
HIF-1*α*/CXCR4						
(1) Both HIF-1*α*/CXCR4 low expression	16 (89)	2 (11)	*P* < .001	18 (100)	0 (0)	*P* = .029
(2) One of HIF-1*α*/CXCR4 high expression	13 (57)	10 (43)	(2) versus (3)	22 (96)	1 (4)	(2) versus (3)
(3) Both HIF-1*α*/CXCR4 high expression	7 (26)	20 (74)	*P* = .028	21 (78)	6 (22)	*P* = .069

HIF-1*α*/VEGF						
(1) Both HIF-1*α*/VEGF low expression	16 (84)	3 (16 )	*P* < .001	19 (100)	0 (0)	*P* = .021
(2) One of HIF-1*α*/VEGF high expression	14 (61)	9 (39)	(2) versus (3)	22 (96)	1 (4)	(2) versus (3)
(3) Both HIF-1*α*/VEGF high expression	6 (23)	20 (77)	*P* = .007	20 (77)	6 (23)	*P* = .062

CXCR4/VEGF						
(1) Both CXCR4/VEGF low expression	15 (94)	1 (6)	*P* < .001	16 (100)	0 (0)	*P* = .012
(2) One of CXCR4/VEGF high expression	13 (65)	7 (35)	(2) versus (3)	100)	0 (0)	(2) versus (3)
(3) Both CXCR4/VEGF high expression	8 (25)	24 (75)	*P* = .004	25 (78)	7 (22)	*P* = .035

HIF-1*α*/CXCR4/VEGF						
(1) Fewer than HIF-1*α*, CXCR4, and VEGF high expression	31 (69)	14 (31)	*P* < .001	44 (98)	1 (2)	*P* < .001
(2) All of HIF-1*α*, CXCR4, and VEGF high expression	5 (22)	18 (78)		17 (74)	6 (26)	
